# Evaluation of the persistence and gene expression of an anti-*Chlamydophila psittaci *DNA vaccine in turkey muscle

**DOI:** 10.1186/1746-6148-2-18

**Published:** 2006-06-09

**Authors:** Karolien Loots, Bart Vleugels, Ellen Ons, Daisy Vanrompay, Bruno Maria Goddeeris

**Affiliations:** 1 Division of Gene Technology, Department of Biosystems, Faculty of Bioscience Engineering, Catholic University Leuven, Kasteelpark Arenberg 30, 3001 Leuven, Belgium; 2 Department of Molecular Biotechnology, Faculty of Bioscience Engineering, Ghent University, Coupure Links 653, 9000 Ghent, Belgium; 3 Laboratory of Veterinary Immunology, Faculty of Veterinary Medicine, Ghent University, Salisburylaan 133, 9820 Merelbeke, Belgium

## Abstract

**Background:**

DNA vaccination has been shown to elicit specific cellular and humoral immune responses to many different agents in a broad variety of species. However, looking at a commercial use, the duration of the immune response against the vaccine is critical. Therefore the persistence of the DNA vaccine, as well as its expression, should be investigated. We conducted these investigations on a DNA vaccine against *Chlamydophila psittaci*, a Gram-negative intracellular bacterium which causes respiratory disease in turkeys and humans. Previous studies showed that the DNA vaccine confers partial protection against *C. psittaci *infection in turkeys. Turkeys were injected intramuscularly with the DNA vaccine : a eukaryotic expression vector (pcDNA1::MOMP) expressing the major outer membrane protein (MOMP) of an avian *C. psittaci *serovar D strain. Over a period of 11 weeks, cellular uptake of the DNA vaccine was examined by PCR, transcription of the insert by reverse transcript-PCR (RT-PCR) and mRNA translation by immunofluorescence staining of muscle biopsies.

**Results:**

The results indicate that the DNA vaccine persists in turkey muscle for at least 10 weeks. Moreover, during this period of time MOMP was continuously expressed, as evidenced by the immunofluorescence staining and RT-PCR.

**Conclusion:**

Since *C. psittaci *infections occur at the age of 3 to 6 and 8 to 12 weeks, a vaccine persistence of 10 weeks seems adequate. Therefore, further research should concentrate on improving the elicited immune response, more specifically the cell-mediated immune response, rather than prolonging the lifespan of the plasmid.

## Background

Genetic vaccination with plasmid DNA expression vectors encoding the sequence of a specific antigen offers a promising and practical approach for the induction of protective immunity. This type of vaccine has been shown to induce a protective immune response against viral [1], bacterial [2] and parasitic [3] diseases in a broad range of species and has several important advantages over commercial vaccines, both subunit and live attenuated. First of all, they induce major histocompatibility complex (MHC) class I restricted CD8+ T-cell responses [4,5]. Secondly, sufficient quantities of plasmid DNA can easily be produced in a relatively cost-effective manner, after which the vaccine can be stored with relative ease [4,6]. Furthermore, DNA vaccines may overcome inherent unresponsiveness in neonatal animals and function in the presence of maternally derived immunity [4].

However, not withstanding these advantages, some reservations for commercial application remain. First, integration of the injected plasmid DNA into the genome of the host cell could occur [5,7,8]. Secondly, repeated injections could lead to an immunological tolerance [9] or induce autoimmunity [10]. Thirdly, injected DNA could induce an immune response against the plasmid backbone [11]. Finally, while the advantage of a long-time persistence probably provides a long-term production and presentation of the protein to the immune system, there is a risk of plasmid DNA residue in the poultry meat used for human consumption. To address these last issues, we studied the persistence and expression of pcDNA1::MOMP, a plasmid DNA expression vector encoding the 'major outer membrane protein' (MOMP) of *Chlamydophila psittaci *serovar D strain 92/1293 [12] after intramuscular injection in commercial turkeys. *C. psittaci*, a Gram-negative obligate intracellular bacterium, is highly prevalent on European turkey farms and causes respiratory infections. This results in substantial economic losses due to expensive antibiotic treatment, weight loss, increased mortality and condemnation at slaughter [13,14]. Up to now, no vaccine is available for *C. psittaci *in birds. However, previous studies have extensively shown the elicited immune response and protection of pcDNA1::MOMP against *C. psittaci *infection in turkeys [15-18]. The duration of the MOMP expression (and consequently the duration of the elicited immune response) after intramuscular injection has not been studied yet.

## Results

### Detection of the plasmid DNA by PCR analysis

**Figure 1:**
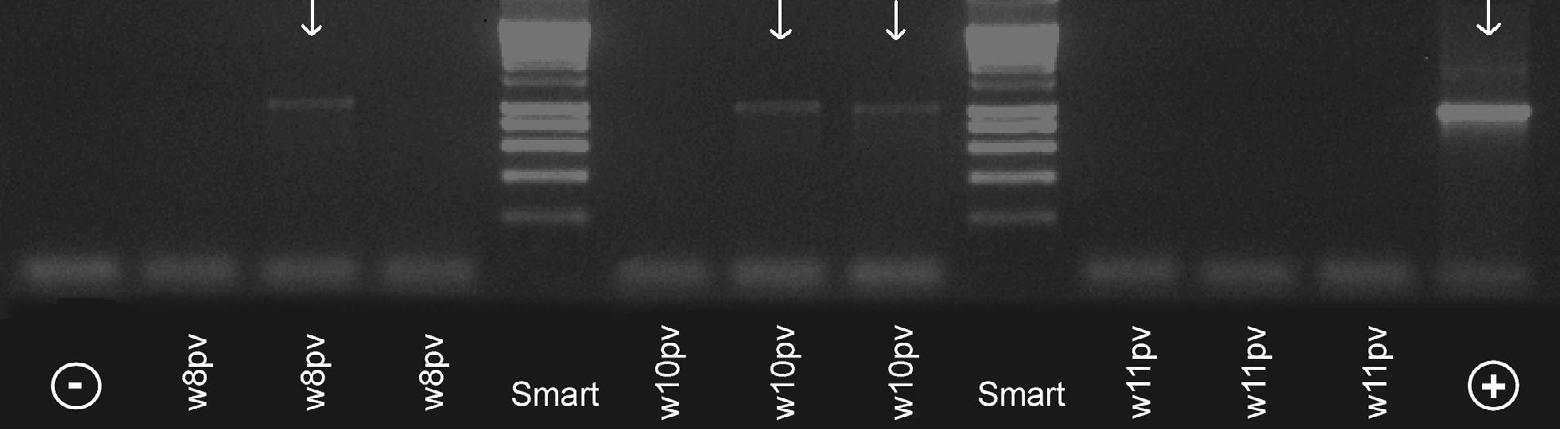
**PCR detection of the plasmid DNA in muscle tissue of vaccinated turkeys**. Gel image representing the PCR-products, obtained by PCR on DNA extracted from muscle tissue at 8, 10 and 11 weeks p.v. (w8pv, w10pv and w11pv). The 3 different samples of the same date, visible on the gel, are 3 DNA extractions from the pooled stock of muscle tissue at the (presumed) injection site, on which the PCR was conducted. The arrows indicate where a band of 1084 bp is visible. The SmartLadder (Eurogentec) was used as molecular size marker (with bands of 200, 400, 600, 800, 1000, 1500, 2000, 2500, 3000, 4000, 5000, 6000, 8000 and 10000 bp). DNA of non-vaccinated muscle tissue was used as a negative control (-) and the pcDNA1::MOMP vector as a positive control (+). At 8 weeks p.v., a band of 1084 bp could still be detected in 1 out of 3 DNA isolates and at 10 weeks p.v. in 2 out of 3 DNA isolates. At 11 weeks p.v. no band could be detected on the ethidium bromide stained gel.

In order to assess the detection limit (sensitivity) of the PCR, a ten-fold dilution series of the plasmid DNA (spiked with turkey muscle DNA) was prepared. The PCR reaction was conducted as described in methods. The results indicated that the lower PCR detection limit was 34.4 fg of plasmid DNA (data not shown). Next, the same PCR reaction was performed on DNA isolates of the tissue samples taken at euthanasia. All PCR results for the DNA isolates of the muscle tissues at the injection site were positive (for the 3 different DNA isolates as well as for the 3 repetitions) up to 7 weeks p.v. At 8 weeks p.v. only 1 out of 3 isolates was positive. At 9 weeks p.v. none of the isolates were positive, even after using a higher amount of plasmid DNA to conduct the PCR. At 10 weeks p.v. two out of 3 isolates were positive. At 11 weeks p.v. none of the isolates were positive. The injected black ink could be detected up to 10 weeks p.v. However, at 11 weeks p.v. the injection site could no longer be visualised by the black ink and sampling became more random (figure 1). No DNA vaccine could be detected in the opposite (non-vaccinated) muscle tissue of the vaccinated turkeys or in the site of the same thigh, but remote from the injection site, during the entire length of the experiment (data not shown).

### OmpA expression analyses

**Figure 2:**
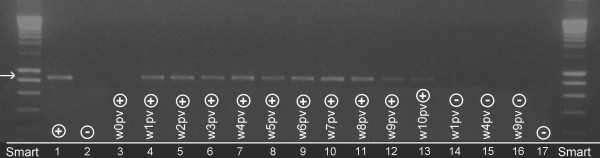
**RT-PCR detection of mRNA in muscle tissue of vaccinated turkeys**. Gel image representing the PCR results on cDNA, obtained from both vaccinated muscle tissue at the (presumed) site of injection from the day of vaccination (w0pv) to 10 weeks p.v. (lanes 3 to 13) and muscle tissue of the opposite (non-vaccinated) thigh on weeks 1, 4 and 9 p.v. (lanes 14 to 16). The SmartLadder (Eurogentec) was used as a molecular size marker (Smart). MilliQ water was used as a negative control (lanes 2 and 17) and cDNA, generated by RT-PCR from mRNA produced *in vitro *from pcDNA1::MOMP, as a positive control (lane 2). A band of 850 bp could be detected by gel electrophoresis from 1 to 10 weeks p.v. after PCR on cDNA from vaccinated muscle tissues, at the (presumed) site of injection (lanes 4 to 13). No PCR products were obtained after performing a PCR on cDNA from muscle tissue of the opposite (non-vaccinated) thighs (lanes 14 to 16) or muscle tissue at the site of injection at the day of vaccination (lane 3).

RNA was isolated from the pooled muscle samples (at the site of injection), as well as from the non-vaccinated muscle tissue and the spleen of the vaccinated turkeys. An RT-PCR was performed 3 times on the same RNA sample. A PCR product was detected in all samples taken at the injection site, until 10 weeks p.v. No PCR product could be detected at the day of vaccination or at 11 weeks p.v. (figure 2 – lanes 3 to 13). No pcDNA1::MOMP-specific mRNA could be detected in the non-vaccinated muscle tissue of the vaccinated turkeys (figure 2 – lanes 14 to 16) nor in the spleen (data not shown).

### *In situ *immunodetection of the expressed MOMP

**Figure 3:**
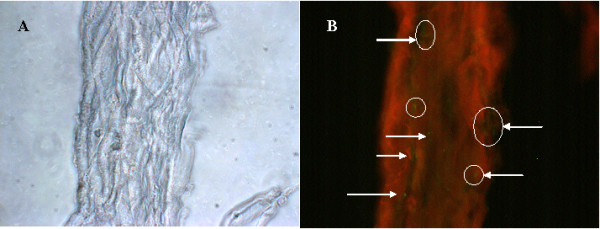
**Detection of rMOMP expression by immunofluorescence staining in muscle tissue of vaccinated turkeys**. The pictures show muscle tissue of vaccinated turkeys, at the site of injection, at 10 weeks p.v., viewed under a microscope under different light conditions (magnified 25 times). Picture 'A' shows the muscle cells under visible light. Picture 'B' shows the presence of green fluorescent rMOMP (by FITC-labelled antibodies, indicated on several places by white arrows) on a red background of muscle cells (Evans' Blue) under light of a wavelength of 488 nm.

There was no rMOMP expression (no fluorescence) detected in the negative control, nor in the non-vaccinated muscle tissue of the other thigh. On the other hand, rMOMP could be detected in all cryosections of the vaccinated muscle tissue up to the end of the experiment, 11 weeks p.v. (data not shown). Figure 3 shows the presence of the stained rMOMP (green fluorescent dots) in muscle tissue (red cells), at the site of injection at 10 weeks p.v.

## Discussion

This study is the first to prove a prolonged presence and activity of the pcDNA1::MOMP vaccine in turkeys. Earlier studies reported a persistence of DNA vaccines ranging from 17 days [19] up to 2 years [20] in various species. Differences in persistence can be attributed to several factors such as the route of administration, the dose, the nature of the antigen, the animal species, etc. Studies have shown that following intravenous, intracerebral or intranasal DNA vaccination, rapid clearance was observed in mice [21,22]. However, following intramuscular (i.m.) or intradermal administration, Chun *et al*. reported persistence up to 8 weeks [22]. Other studies also showed a increased persistence in mice (8 months) in comparison to chickens (17 days) after i.m. immunization with the same amount of plasmid [19]. The latter can be explained by the growth rate and size of the animals, as correct tissue sampling in chickens becomes more difficult as the birds grow larger. In the current experiment, tissue selection was facilitated by simultaneous injection of black ink. This allowed a more precise selection of the inoculated tissues, which could explain the prolonged persistence of the DNA vaccine used in this experiment.. At 10 weeks p.v. however, the simultaneously injected black ink was no longer visible. Probably, at this time, there was no more DNA vaccine detection because the site of injection could not be retrieved. Although there are currently no data available suggesting a correlation between the administered dose and the persistence, the four-fold higher dose used in the current experiment compared to the dose used by Morris-Downes *et al*. could contribute to the longer persistence [19]. Moreover, in our experiment, higher injection volumes were used.  Earlier studies showed that higher hydrostatic pressure, resulting from a higher injection volume, enhanced the uptake of plasmid DNA by muscle cells [23]. Furthermore, Morris-Downes *et al*. suggested that the nature of the antigen and its ability to induce a strong immune response could influence its persistence [19]. Next, the choice of the promotor could also have an effect on gene expression, since Davis *et al*. discovered a 1000-fold increase in gene expression when comparing the Rous Sarcoma Virus (RSV) promotor to the Simian Virus 40 (SV40) promoter [24]. Finally, in 4 to 6 week-old mice a higher gene expression was observed in comparison to 10 week-old mice [25], so the age of the animals (and consequently the growth rate) can also play a role in the persistence.

## Conclusion

Since the lifespan of commercial turkeys is about 15 weeks and most *C. psittaci *infections occur at the age of 3 to 6 and 8 to 12 weeks [26], a persistence of 10 weeks seems adequate. Secondly, in view of social hesitance concerning DNA vaccination and the resulting transgenic animals, DNA vaccination should be carefully investigated. Finally, previous experiments in 'specific pathogen free' (SPF) turkeys already showed partial protection against *C. psittaci *infection by pcDNA1::MOMP vaccination. Therefore, the main focus of further research should be on improving the elicited immune response, more specifically the cell-mediated immune response, rather than prolonging the lifespan of the plasmid.

## Methods

### 2.1. Experimental set-up

The experimental design was evaluated and approved by the Ethical Commission for Animal Experiments of the K.U.Leuven. Fifty five 1-day old commercial 'Big 6' turkeys were housed in open pens under a heating and lighting scheme as in conventional rearing and received food and water *ad libitum*. Plasmid pcDNA1::MOMP was constructed by sticky-end ligation of the *outer membrane protein A *(*ompA*) gene of *C. psittaci *serovar D strain 92/1293 into the *Eco*RI site of pcDNA1 (Invitrogen) and purified and quantified as described previously [12]. At 10 days of age, turkeys were injected with a single dose of 100 μg of pcDNA1::MOMP dissolved in 0,9% saline solution and 1% black Indian ink (Pelikan) in a total volume of 300 μl into the central portion of the left thigh muscle (*m. quadriceps*). From 1 to 11 weeks post vaccination (p.v.), five turkeys were euthanized each week. At the time of euthanasia, 4 different tissue samples were taken from each animal. One sample from the muscle of the vaccinated thigh, located near the black ink; another sample from the muscle of the vaccinated thigh, but well away from the ink. A third sample was taken from the non-vaccinated thigh and finally, the spleen was collected. Tissues from the same location, taken at the same day, were pooled.

### 2.2. Detection of the plasmid DNA by PCR analysis

Total DNA was extracted from 25 mg of the pooled muscle samples by means of the QIAamp DNA Mini Kit (QIAGEN). For each time of euthanasia, the samples from the same tissue (position) were pooled. On that pool 3 DNA isolations were performed. For each DNA isolate, the concentration and purity was checked by measuring the optical density (OD) at 260 nm and the OD(260 nm)/OD(280 nm) respectively. Next, a PCR was performed on 100 ng of plasmid DNA, for each of the 3 DNA isolates. For reproducibility, each PCR was performed 3 times. The PCR reaction mixture contained 50 mM KCl, 20 mM Tris-HCl (pH 8.3), 2 mM MgCl_2_, 0.1% Tween20, 200 μM of each dNTP, 1.25 μM of each primer, SuperTaq buffer (10x), 0.1 unit SuperTaq polymerase (5U/μl) and 100 ng of plasmid DNA in a total volume of 50 μl. After an initial denaturation at 95°C for 5 minutes, 35 cycles of one min at 95°C, two minutes at 55°C and three minutes at 72°C, with a final extension at 72°C for 5 minutes, were performed. The used primers were the commercial primer T7 (5'-AATACGACTCACTATAGGG – 3'), located on the pcDNA1 vector, and EXR05 (5'-TGCTAGACCAACTTGCCATT – 3'), located on the inserted *ompA *gene. This provided a means to distinguish between the presence of the DNA vaccine and an unexpected *Chlamydophila *infection. Amplification products should have a size of 1042 base pairs (bp). Before analysis of the samples, the sensitivity of the PCR set up was determined by performing the PCR on dilution series of the DNA vaccine spiked with genomic DNA of muscle tissue.

### 2.3. OmpA expression analyses

Total RNA was extracted from the pooled muscle samples by TRIzol™ (Invitrogen), a commercial protocol based on the method of Chomczynski and Sacchi (1987), but with an extra DNase treatment to remove plasmid and genomic DNA. For each RNA isolate, the concentration and purity was checked by measuring the OD at 260 nm and the OD(260 nm)/OD(280 nm) respectively. cDNA was generated from the RNA template using primer EXR02 (5'-GGTTGAGCAATGCGGATAGTAT-3') and Omniscript Reverse Transcriptase according to the QIAGEN RT-PCR System protocol. The cDNA was then amplified by PCR using the primers EXF03 (5'-CCTGTAGGGAACCCAGCTAGAA-3') and EXR02, yielding a 850-bp product. Both EXR02 and EXF03 are located on the *ompA *gene. The PCR reaction was performed in a total volume of 20 μl containing Taq buffer (10x), 2.5 units Taq polymerase (5U/μl), 2 mM MgCl_2_, 1 μM of each primer and 0.2 μM of each dNTP. After an initial denaturation at 95°C for 5 minutes, 35 cycli were performed accordingly: 3 minutes at 95°C, 1 min at 51°C and 0.5 min at 72°C, followed by a final extension at 72°C for 5 minutes.

### 2.4. In situ immunodetection of the expressed MOMP

Four different cryostat sections (10 μm) of both the muscle tissue at the site of injection and the non-vaccinated muscle tissue of the other thigh were prepared for each day of euthanasia. As a negative control, cryostat sections of the muscle tissue of non-vaccinated animals were used. The sections were examined by an indirect immunofluorescence staining as described previously [12], using a monoclonal antibody against a genus-specific epitope on the MOMP of *Chlamydiaceae*.

## Competing interests

The author(s) declare that they have no competing interests.

## Authors' contributions

KL was involved in conception and design of the experiment and was responsible for the organization and execution of the experiment, interpretation of the data and writing of the report.

BV and EO assisted in analysis of the laboratory data, interpretation of the data and writing of the report.

DV and BMG were project leaders.
